# Family-based interventions for substance misuse: a systematic review of systematic reviews—protocol

**DOI:** 10.1186/2046-4053-3-90

**Published:** 2014-08-15

**Authors:** Yasmin Akram, Alex Copello, David Moore

**Affiliations:** 1 University of Birmingham, Birmingham B15 2TT, UK; 2 Birmingham and Solihull Mental Health Foundation Trust, Birmingham B31 5JE, UK

**Keywords:** Families, Family therapy, Systematic review, Psychological interventions, Family interventions, Alcohol misuse, Drug misuse

## Abstract

**Background:**

Worldwide, there are an estimated 15 million individuals with drug use disorders and over five times as many with alcohol use disorders (WHO 1:2, 2005). Most individuals with substance misuse have families who are affected. Initial scoping searches identified an expanse of broad and disparate studies and reviews on the family interventions for substance misuse. This systematic review of systematic reviews aims to bring together the expanse of research on the effectiveness of family-based interventions in substance misuse.

Initial scoping searches identified an expanse of broad and disparate studies and reviews on the family interventions for substance misuse. This systematic review of systematic reviews aims to bring together the expanse of research on the effectiveness of family-based interventions in substance misuse.

**Methods:**

Extensive electronic and manual searches will be undertaken. Screening, data extraction and quality assessment will be undertaken by two reviewers with disagreements resolved through discussion.

The inclusion criteria will be that the study is a systematically undertaken review, the population is individuals with substance misuse problems and the interventions include a family-focused component. Reviews that focus on prevention rather than treatment will be excluded.

The reviews will be assessed for quality and relevance. The evidence from included systematic reviews will be mapped by focus of intervention (promoting engagement of user into treatment/joint involvement in treatment of user/treating family member in own right) for both adults and adolescents for drug and/or alcohol misusers to allow assessment of the density of available evidence. The higher-quality, up-to-date evidence for each domain will be identified and described, and conclusions will be drawn with limitations of the evidence highlighted.

**Discussion:**

This systematic review of systematic reviews will be an efficient and robust way of looking at the current state of the evidence in the field of family-based interventions for substance misuse.

It will evaluate all the available systematic-review-level literature to report on the effectiveness of family-based psychological interventions in improving substance-related outcomes and improving health and wellbeing of substance misusers and/or their families. This will inform future treatment policies and commissioning decisions.

In addition, it will identify areas of poor quality, inconsistency and gaps in the evidence base for family-based psychological interventions in substance misuse with respect to secondary evidence in order to inform future research.

**Systematic review registration:**

PROSPERO CRD42014006834

## Background

Worldwide, there are an estimated 15 million people with drug use disorders and 76 million with alcohol use disorders [1].

Most individuals with substance misuse have family and significant others who are affected by the problem user regardless of whether they have become estranged or remain close and even provide caring roles.

Substance misuse affects both the physical and psychological wellbeing of family members resulting in greater diagnoses of depression, substance misuse and increased use of healthcare services compared to similarly matched controls [2].

Family members are also at higher risk of exposure to antisocial and criminal behaviours from the substance misuser [3], in addition to other substance-misuse-related problems such as poor mental health, unemployment, deprivation, marriage problems, domestic abuse and affected parental capacity [4,5].

During recent years, the importance of providing support to affected family members, in addition to the key role these individuals can play in improving the substance-related outcomes for the user, has become increasingly salient [6,7].

A range of behavioural interventions incorporating a family component into treatment have been developed and empirically tested, and these can be broadly grouped into three categories [8]:

• ‘Working with family members to promote the entry and engagement of substance misusers into treatment’

• ‘Joint involvement of family members and substance-misusing relatives in the treatment of the latter’

• ‘Interventions responding to the needs of the family members in their own right’

Many studies on these interventions have been undertaken. The current literature is broad with individual studies and reviews focusing on selected subgroups of users, interventions or outcomes. Several systematic reviews have been published in the field each with varying population and outcome focuses [9-12].

This systematic review of systematic reviews aims to bring together the expanse of research on the effectiveness of family-based interventions in substance misuse, highlighting the strength and quality of the evidence available across the field, key themes and implications for practice and areas for further research.

## Aims and objectives

### Aim

This systematic review of systematic reviews aims to summarise evidence from multiple systematic reviews of various family-based interventions for substance misuse including alcohol and illicit drugs.

### Objectives

This systematic review of systematic reviews sets out:

• To undertake a systematic review of the available systematic reviews on psychological interventions involving the families of substance misusers

• To evaluate through the above process the available literature on the effectiveness of family-based psychological interventions in improving substance-related outcomes and improving health and wellbeing of substance misusers and/or their families

• To identify areas of weakness, inconsistency and gaps in the evidence base for family-based psychological interventions in substance misuse in terms of secondary evidence in order to inform future research

• To develop evidence for dissemination to public health and drugs and alcohol teams (DAAT) in order to inform substance misuse commissioning decisions

## Methods/Design

The methodology will involve the following steps:

• A systematic literature search for systematic reviews (published and within the grey literature): search strategy

• Objective selection of relevant systematic and meta-analytic reviews: screening and selection

• Critical appraisal of the included systematic reviews: data appraisal

• A structured synthesis of the research findings: data analysis

Details of these steps are described below.

### Search strategy

The search aims to identify all systematic reviews assessing the effectiveness of family-based interventions in substance misuse.

Relevant terms for the family therapies/interventions were derived from the initial scoping of literature. A comprehensive search strategy used in a previous review will be used as a reference and expanded and modified to suit the requirements of this systematic review [13]. In particular, the search will be extended to include drug misuse in addition to alcohol misuse, and a greater number of synonyms with respect to the keywords will be employed in order to make the search as comprehensive as possible.

The search strategy will be adapted depending on the unique requirements of each individual database in order to ensure that the most efficient and relevant search is performed. A dual strategy employing a combination of Medical Subject Headings (MeSH) and ‘free-text’ terms will be used where possible to ensure maximum coverage.

In each database, all family intervention terms (each combined with an ‘OR’) will then be combined with all alcohol and drug terms (each combined with an ‘OR’).

The following electronic databases will be searched:

• Cochrane Library (CDSR, DARE, HTA, CENTRAL)

• Campbell Collaboration

• MEDLINE (Medical Literature Analysis and Retrieval System Online; indexed and non-indexed)

• Embase

• CINAHL (Cumulative Index to Nursing and Allied Health Literature)

• PsycINFO (Database of Psychological Literature)

• IBSS (International Bibliography of Social Sciences)

• CPCI (Conference Proceedings Citation Index)

Other sources to be searched to identify additional literature include:

• SIGLE (System for Information on Grey Literature in Europe)

• PROSPERO (for ongoing reviews)

• Citation lists of included systematic reviews

• Contacting authors of ongoing reviews (where a protocol has been identified through the electronic database search, e.g. PROSPERO, but where the full paper does not appear to be published)

There will be no date or language restrictions placed on the searches.

Validated Haynes filters [14] will be used where possible to limit the searches using the clinical queries ‘reviews’ option since this will retrieve systematic reviews, meta-analyses and health technology assessments. The ‘maximises sensitivity’ option will be used in order to maximise the yield and ensure all relevant reviews are obtained. An example search strategy is included in Additional file 1.

Search results will be entered into reference management software (RefWorks). An inbuilt algorithm will be used to identify duplicate records. The databases will additionally be manually checked for any further duplicates.

### Screening and selection

Studies will be selected according to eligibility criteria established a priori using a two-step screening process. Sufficient portions of foreign language reviews will be translated to facilitate selection decisions.

Initially, each title and abstract will be screened by one reviewer to decide whether the full paper should be retrieved, and a random sample of 20% of the citations will be independently checked by a second reviewer. If the checker is identifying more studies to be put through than the original reviewer (who is reviewing all the articles), which on discussion do go through, then the checking will be increased to 100%. If however the checker is identifying additional studies that on discussion are agreed should not be put through, then checking will remain at 20%. All disagreements will go to a third person.

A paper will be excluded at this stage if it is clear from the title and abstract that it does not represent a review or if it does not include a substance misuse or family intervention component. All other citations will be retained for retrieval of full-text articles, which will subsequently be reviewed using the structured inclusion/exclusion form independently by two reviewers with recourse to a third reviewer if necessary.

The screening process will be piloted and revised if necessary.

### Inclusion criteria

A structured pro forma containing the following criteria will be applied to the full copy of the articles:

#### Population

Any misusers of drugs and/or alcohol as defined by the review papers or their families.

#### Intervention

Family-based psychological interventions.

A broad definition of ‘family’ will be used to include spouse, partner, grandparents, parents, sibling, child or concerned significant others in order to make this review as comprehensive as possible.

Similarly, a broad definition of ‘psychological intervention’ as any formal structured psychological or social intervention as defined by the review papers, rather than advice and information, or drug-based treatment.

#### Comparator

No specific comparator column will be employed. All comparators selected for inclusion within the systematic reviews are relevant. These will include interventions targeting only the substance misuser, no current treatment, waiting list controls and alternative family-based interventions.

#### Outcomes

The review must report on the effectiveness of family interventions based on one or more of the following outcomes:

• Primary: Reduction or cessation in substance misuse

• Secondary: Quality of life; behavioural, relationship or family functioning; hospitalisation; mental health; or other relevant outcomes identified by the reviews with respect to the substance misuser and/or the family member

In essence, a broad approach is being taken with respect to outcomes.

#### Study type

Systematic reviews using the definition of a systematic review as per Moher et al. [15] will be included.

The criteria for inclusion as a systematic review will be as follows:

• A statement of review

• A documented search strategy of at least one database with search terms stated (however minimal)

• Stated inclusion/exclusion criteria (however minimal)

In essence, a broad approach will be taken to ensure all relevant reviews are captured.

### Exclusion criteria

The full-text articles will be excluded if

• They focus on prevention of substance misuse rather than treatment.

• If they have a broader approach than the current systematic review of reviews but do not provide a specific systematic sub-analysis relevant to the current systematic review of systematic reviews, e.g. the full-text paper considers families of adolescents involved in criminal behaviour but does not provide a sub-analysis with respect to adolescents with substance misuse problems.

• Where a relevant systematic review is ongoing at the time searches are undertaken and/or published after the searches, it will be noted in the final manuscript but not included in the analyses.

The excluded papers will be listed with the reason for exclusion noted.

## Data appraisal

### Data mapping

The reviews will be mapped by focus of intervention against the substance of misuse for both adults and adolescents to allow assessment of the density of available evidence.

A) Reviews looking at the following three areas

1. Working with family members to promote the entry and engagement of substance misusers into treatment

2. Joint involvement of family members and substance-misusing relatives in the treatment of the latter

3. Interventions responding to the needs of the family members in their own right

B) Will be explored for both

1. Adolescent

2. Adult

C) Substance misusers who fall into each of the following categories

1. Alcohol misusers

2. Drug misusers

3. Misusers of both alcohol and drugs

Thus, 18 different domains (A × B × C) will be explored. Reviews within each domain (e.g. reviews looking at joint involvement of family in the treatment of adult alcohol misusers) will be described and assessed for quality and relevance. Where a systematic review includes studies across more than one category, it will be included in all relevant domains.

The systematic review papers within each domain will be assessed in a stepwise fashion based on the following three characteristics in order to report on the best available evidence in each domain:

1. Periodicity

2. Quality

3. Reporting

Systematic reviews will only be excluded from further scrutiny where there is clearly more appropriate evidence based on these assessment criteria. These characteristics are described more fully below.

There will always be a certain degree of subjectivity whilst applying the above criteria; however, by consistently applying the criteria across each of the domains, comparisons between the quality and relevance of available evidence in each domain can be reliably made.

### Periodicity

There are no validated criteria for when to update a systematic review; however, an assessment can be made on the basis of when the searches were conducted and how fast moving the field is [16]. Over the past decade, a significant amount of research on family interventions in substance misuse has been undertaken.

The following questions will be considered in making a judgement about the periodicity of the systematic reviews:

• When were the searches done?

• Is newer research likely to have affected the conclusions that can be drawn from this research?

• Have changes in clinical practice affected the relevance of the data?

Thus, for example, if in the domain looking at joint involvement of family in the treatment of adult alcohol misusers five systematic reviews are found, however two include searches over 15 years old and describe treatments that are no longer used in practice, but the remaining three have searches within the last 5 years only the latter three will be analysed further.

### Quality assessment of reviews

The quality of the remaining systematic reviews in each domain will be assessed by one reviewer and independently checked for accuracy by a second reviewer using the ‘A Measurement Tool to Assess Systematic Reviews’ (AMSTAR) tool [17].

This will allow an assessment of what the quality of the most up-to-date and relevant evidence in each domain is and allow conclusions to be drawn in the context of the best-quality evidence. For example, if after application of the above steps three reviews remain in the domain looking at joint involvement of family in the treatment of adult alcohol misusers but one in particular performs poorly in several assessment areas, e.g. search strategy, quality assessment of included studies and publication bias, then only the other two reviews will be used to draw evidence from.

### Reporting of reviews

The remaining reviews will be checked for their adherence to the Preferred Reporting Items for Systematic Reviews and Meta-Analyses (PRISMA) reporting guidelines for systematic reviews to see whether the conclusions that can be drawn from the paper are affected by reporting issues [15]. No further reviews will be excluded at this stage; however, this assessment will allow recommendations for future systematic reviews in each domain to be made.

Whilst the above process is by nature subjective by having a clear, structured approach and being transparent about decisions made, any bias will be explicit.

### Data extraction

For included reviews, summary data relating to review characteristics, interventions and results will be extracted and collated into structured tables by one reviewer and independently checked by a second reviewer. The following details will be extracted:

• Author

• Year of publication

• Country

• Title

• Primary focus of review e.g. family intervention, substance misuse

• Review question in terms of

○ Number of included studies

○ Population e.g. adults, adolescents

○ Type of family therapy e.g. joint involvement

○ Interventions included e.g. couples therapy

○ Outcomes assessed e.g. cessation of drug use

• Summary of results

• Issues raised with respect to the review

○ Quality factors

○ Reporting factors

The primary studies included within each review will be tabulated in order to consider the contributing evidence and level of overlap between the reviews.

### Data analysis

A narrative approach will be used to describe the evidence. The level of evidence supporting the interventions for each available outcome in each domain of interest will be described e.g. the evidence for improved relationship satisfaction in adult alcohol misusers following joint involvement in treatment using couples will be described.

Care will be taken to ensure that where there is overlap between the primary studies included within the systematic reviews, this is noted and considered when drawing conclusions from the evidence. The limitations of the data and gaps in the evidence for each domain will also be highlighted.

Following this, a higher-level analysis will be undertaken to see whether there are general consistencies across domains that can be identified and conclusions drawn with respect to the evidence of for different family-based interventions in substance misusers in general.

### Data reporting

The protocol will be registered on the PROSPERO database, and the systematic review reported according to PRISMA guidelines.

### Dissemination plans

The findings will be submitted for publication in one or more peer-reviewed journals. They will also be disseminated through appropriate media, e.g. conferences/meetings to substance misuse advisory teams, treatment commissioners and providers and wider interested audiences.

## Discussion

This systematic review of systematic reviews will be an efficient and robust way of looking at the current state of the evidence in the field of family-based interventions for substance misuse.

The strengths of this systematic review of systematic reviews will lie in its systematic nature and broad, comprehensive search strategy, quality assessment and data analysis. Through this process, all the available secondary literature will be evaluated in order to report on the effectiveness of family-based psychological interventions in improving substance-related outcomes and improving health and wellbeing of substance misusers and/or their families. This will inform future treatment policies and commissioning decisions.

In addition, it will identify areas of weakness, inconsistency and gaps in the evidence base for family-based psychological interventions in substance misuse with respect to secondary evidence in order to inform future research. The work will highlight both areas where well-conducted systematic reviews have identified weaknesses of the primary evidence, in addition to where there are weaknesses of the secondary evidence, in terms of poor-quality systematic reviews.

## Abbreviations

AMSTAR: A Measurement Tool to Assess Systematic Reviews; CDSR: Cochrane Database of Systematic Reviews; CENTRAL: Cochrane Central Register of Controlled Trials; CINAHL: Cumulative Index to Nursing and Allied Health Literature; CPCI: Conference Proceedings Citation Index; DAAT: drugs and alcohol team; DARE: Database of Abstracts of Reviews of Effects; IBSS: International Bibliography of Social Sciences; MEDLINE: Medical Literature Analysis and Retrieval System Online; MeSH: Medical Subject Headings; PRISMA: Preferred Reporting Items for Systematic Reviews and Meta-Analyses; PsycINFO: Database of Psychological Literature.

## Competing interests

The authors declare that they have no competing interests.

## Authors' contributions

AC helped conceive the study, participated in the design and coordination of the study, and was involved in revising the manuscript critically for important intellectual content. DM participated in the design of the study and was involved in revising the manuscript critically for important intellectual content. YA helped conceive the study, participated in the design and coordination of the study, carried out the scoping search and drafted the study manuscript. All authors read and approved the final manuscript.

## Supplementary Material

Additional file 1 **Example search strategy.** This gives details of the search strategy run in a particular database (MEDLINE). Click here for file
