# Supplementary material for: Family-based interventions for substance misuse: a systematic review of systematic reviews—protocol
Source: Syst Rev. 2014 Aug 15;3:90. doi: 10.1186/2046-4053-3-90 (PMC4150116; doi:10.1186/2046-4053-3-90)
Supplement: Additional file 1 — Example search strategy. This gives details of the search strategy run in a particular database (MEDLINE). [file 2046-4053-3-90-S1.docx]

**Additional File 1. Example Search Strategy**

**Database: Ovid MEDLINE (R) 1966 to April Week 3 2014**

1. couples therapy.mp. or exp Couples Therapy/

2. family therapy.mp. or exp Family Therapy/

3. conjoint therapy.mp.

4. cooperative council.mp.

5. network therapy.mp.

6. marital therapy.mp. or exp Marital Therapy/

7. (helping adj2 family).mp.

8. (Twelve step facilitation).mp.

9. (five step intervention).mp.

10. families anonymous.mp.

11. (community reinforcement adj1 family training).mp.

12. Johnson institution intervention.mp.

13. unilateral family therapy.mp.

14. multidimensional family therapy.mp.

15. (pressure adj1 change).mp.

16. behaviour$ couple$ therapy.mp.

17. behavior$ couple$ therapy.mp.

18. family systems therapy.mp.

19. (social behaviour adj1 network therapy).mp.

20. (social behavior adj1 network therapy).mp.

21. behaviour$ exchange$ system.mp.

22. behavior$ exchange$ system.mp.

23. (mutual$ adj2 help$).mp.

24. relational intervention$.mp.

25. ((parent$ or mother$ or maternal or father$ or paternal or sibling$ or family) adj2 (relation$ or intervention$ or skill$ or training$ or therap$)).mp.

26. or/1-25

27. alcohol related disorders.mp. or exp Alcoholism/ or exp Alcohol-Related Disorders/ or exp Drinking Behavior

28. drug-related disorders.mp. or exp Substance-Related Disorders

29. ((alcohol$ or drug$ or substance$) adj2 (disorder$ or addiction$ or use$ or misuse or abuse$ or excess or consumption or drink$ or dependen$)).mp.

30. or/27-29

31. 26 and 30

32. limit 31 to "reviews (maximizes sensitivity)"
